# Supplementary material for: Influence of psychological capital on core competency for new nurses
Source: PLoS One. 2023 Aug 10;18(8):e0289105. doi: 10.1371/journal.pone.0289105 (PMC10414633; doi:10.1371/journal.pone.0289105)
Supplement: Attachment — Submitted filename: Response to Reviewers.docx [file pone.0289105.s003.docx]

Dear Editor and Reviewers:

Thank you for your comments concerning our manuscript entitled “Influence of psychological capital on core competency for new nurses”. Those comments are all valuable and very helpful for revising and improving our paper, as well as the important guiding significance to our research. We have studied the comments carefully and have made corrections which we hope meet with approval. Revised portions are highlighted on the paper. The main corrections in the paper and the responses to the reviewer’s comments are as flowing:

Response to the reviewer’s comments:

Reviewer #1:

1. Response to comment: The manuscript needs to be strictly followed and the instructions returned by the journal.

Response: We have adjusted the manuscript format to comply with the journal's requirements as per your suggestion.

2. Response to comment: The abstract's content needs to be reorganized. Please describe the sorts of ANOVA analysis that were employed in the method.

Response: We have reorganized the abstract's content and provided a description of the one-way ANOVA analysis used in the methodology.

3. Response to comment: Additional information is needed in the introduction.

Response: Additional information has been added to the introduction section, focusing on the development of core competency and psychological capital in new nurses. We have highlighted the changes made, emphasizing the importance of understanding factors such as challenges during the transition, organizational culture, leadership support, workload, and interpersonal relationships..

4. Response to comment: The methodology requires extra info to make robust.

Response: We included all new nurses entering in 2020-2022 and did not calculate a sample size. We further stated in the study limitations that the use of convenience sampling was motivated by accessibility and usability considerations and that alternative sampling methods, such as random sampling, could be discussed and considered for better representation of the target population. It is recommended that the results be considered as preliminary findings and future studies are encouraged to validate them on a larger sample and more representative sample. In addition, we have added a description of the validity and reliability of the originality of the questionnaire, as well as a description of the scoring system and the detailed domains.

5. Response to comment: The explanation on the application of the hierarchy multiple regression analysis

Response: We have provided a more specific explanation of the hierarchical multiple regression analysis, highlighting its relevance and implications.

6. Response to comment: Table 1 – how did you define the “new nurses” when you include those who already three years in service? Nature of the nurses – how did you define it? What is the difference between contract nurses and staff nurses?

Response: The definition of a new nurse may vary by country, region and healthcare institution. In China, nurses are often required to undergo three years of nursing training, including theoretical studies and practical training. Therefore, defining nurses within three years of training as new nurses may reflect their status during the training cycle. Staff nurses are permanent employees of healthcare facilities, enjoying benefits, job security, and career opportunities. Contract nurses, however, work temporarily and may have less job security and benefits. In some cases, contract nurses may perform similar duties as staff nurses but with different employment arrangements. To reduce ambiguity, we changed the staff nurse to a permanent nurse

7. Response to comment: Modifications to table 2

Response: We added reporting on the minimum and maximum values. The total dimensional score reflects the overall score of the participants on each dimension, and it helps us to understand the performance of the participants on the whole scale. And the dimensional mean scores allow us to understand the average level of participants' performance on each dimension by calculating the mean of the scores on each dimension.

8. Response to comment: Modifications to table 3

Response: e have indicated that "p" represents the 1% level of significance in Table 3.

9. Response to comment: Modifications to table 4

Response: We have made revisions to Table 4, redescribed the section on hierarchical multiple regression analysis, and modified the corresponding discussion.

Reviewer #2:

1. Response to comment: Underscore the significance and distinctiveness of this paper.

Response: We have emphasized the significance and distinctiveness of our paper in the discussion section, highlighting the focus on examining the relationship between psychological capital and core competency and the fresh perspective it offers within the field.

2. Response to comment: Augmenting the existing information or introducing research questions/research hypotheses in the literature section

Response: Four related hypotheses have been added to the literature section, augmenting the existing information.

3. Response to comment: Inclusion of a research framework within your study

Response: The research framework has been included after the introduction, as per your suggestion.

Reviewer #3:

1. Response to comment: Explain the rationale for researching the effect of psychological capital on core competency and the effect of psychological capital should be investigated.

Response: We have further explained the rationale for researching the effect of psychological capital on core competency and its importance in nursing professionals' development. The purpose and significance of the study have been elaborated upon in the introduction section.

2. Response to comment: The second criterion specified as the selection criterion of the participants is not clear

Response: The selection criterion for participants has been clarified, stating that "new nurses" are those who have no prior work experience as a registered nurse or in any other healthcare-related role.

3. Response to comment: The mean and standard deviations of the study times of the participants. Proof of the "new nurses" designation should have been added.

Response: The definition of a new nurse may vary by country, region and healthcare institution. In China, nurses are often required to undergo three years of nursing training, including theoretical studies and practical training. Therefore, defining nurses within three years of training as new nurses may reflect their status during the training cycle. In China, the new graduate nurses every entry period is July-August. The shortest duration of work for the population in this study was 6 months (34.06%) and the longest was 2.5 years (41.30%). The mean of the working time of the population was 1.23 (SD 0.80).

4. Response to comment: Power analysis is also recommended. It is not clear whether the Cronbach's α values given for the scales used belong to the current study.

Response: While a power analysis was not necessary due to considering the entire population, we have acknowledged the limitations of convenience sampling and suggested alternative sampling methods for future studies. The validity and reliability of the questionnaire have been described, and the scoring system and detailed domains have been provided.

5. Response to comment: Ethics committee approval is indicated, but information on how and how informed consent was obtained should be included.

Response: We have added information on how informed consent was obtained, ensuring participants' voluntary participation without adverse consequences.

6. Response to comment: Hierarchical preference of regression analysis is not appropriate in the analysis of data.

Response: The section on hierarchical multiple regression analysis has been further refined, addressing your concern. Table 4 has been revised accordingly.

7. Response to comment: In line with the purpose of the study, there is a need to deepen the discussion.

Response: We have deepened the discussion section, highlighting the focus of our study on examining the relationship between psychological capital and core competency and the potential influence of psychological resources on nursing competence.

We believe that these revisions have significantly enhanced the clarity, accuracy, and overall quality of our manuscript. We are grateful for your guidance, which has undoubtedly strengthened our research. If you have any further suggestions or require additional information, please do not hesitate to let us know.

Thank you once again for your time and invaluable input. We look forward to the opportunity to submit the revised manuscript.

Best regards,

Mengjie Xia
